# Metamorphic devolatilization of subducted marine sediments and the transport of volatiles into the Earth's mantle

**DOI:** 10.1038/35077056

**Published:** 2001-05-01

**Authors:** D. M. Kerrick, J. A. D. Connolly

**Affiliations:** 1 Department of Geosciences, The Pennsylvania State University, University Park, 16802 Pennsylvania USA; 2 Earth Sciences Department, Swiss Federal Institute of Technology, Zurich, 8092 Switzerland

## Abstract

Volatiles, most notably CO_2_, are recycled back into the Earth's interior at subduction zones^1,2^. The amount of CO_2_ emitted from arc volcanism appears to be less than that subducted, which implies that a significant amount of CO_2_ either is released before reaching the depth at which arc magmas are generated or is subducted to deeper depths. Few high-pressure experimental studies^3,4,5^ have addressed this problem and therefore metamorphic decarbonation in subduction zones remains largely unquantified, despite its importance to arc magmatism, palaeoatmospheric CO_2_ concentrations and the global carbon cycle^6^. Here we present computed phase equilibria to quantify the evolution of CO_2_ and H_2_O through the subduction-zone metamorphism of carbonate-bearing marine sediments (which are considered to be a major source for CO_2_ released by arc volcanoes^6^). Our analysis indicates that siliceous limestones undergo negligible devolatilization under subduction-zone conditions. Along high-temperature geotherms clay-rich marls completely devolatilize before reaching the depths at which arc magmatism is generated, but along low-temperature geotherms, they undergo virtually no devolatilization. And from 80 to 180 km depth, little devolatilization occurs for all carbonate-bearing marine sediments. Infiltration of H_2_O-rich fluids therefore seems essential to promote subarc decarbonation of most marine sediments. In the absence of such infiltration, volatiles retained within marine sediments may explain the apparent discrepancy between subducted and volcanic volatile fluxes and represent a mechanism for return of carbon to the Earth's mantle.

## Main

A premise of our work is that realistic modelling of metamorphic devolatilization of subducted lithologies is only possible on the basis of phase equilibria in chemical systems closely approximating actual bulk compositions. Our studies on metamorphic devolatilization of the other two main carbonate-bearing lithologies involved in subuction zones (ophicarbonates and metabasalts) are considered elsewhere^2,7^. Carbonate is abundant in two main pelagic marine sediment lithologies^8^: (1) siliceous limestones and (2) clay-carbonates (marls). From the database of ref. 8 (see Supplementary Information) we selected bulk compositions of siliceous limestones from the Marianas and Vanuatu trenches, a marl from the Antilles trench and their average marine sediment bulk composition (denoted ‘GLOSS’ in ref. 8). Our computations account for the oxide components: SiO_2_, Al_2_O_3_, FeO, MgO, CaO, Na_2_O, K_2_O, CO_2_ and H_2_O.

**Figure 1:**
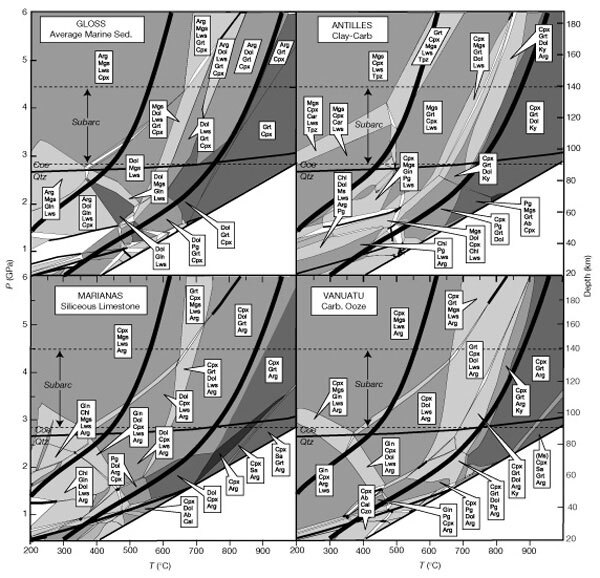
Phase equilibria computed for selected bulk compositions from the database of ref. 8. Further details are available in Supplementary Information. The computer programs, and the thermodynamic data and equations of state utilized by these programs, are available at http://www.erdw.ethz.ch/∼jamie/perplex.html. Mineral abbreviations are: Ab, albite; Arg, aragonite; Cal, calcite; Car, Mg-Fe carpholite; Chl, chlorite; Coe, coesite; Cpx, clinopyroxene; Czo, clinozoisite; Dol, dolomite; Gln, glaucophane; Grt, Garnet; Ky, kyanite; Lws, lawsonite; Mgs, magnesite; Pg, paragonite; Qtz, quartz; Sa, Sanidine; Tpz, topaz. With the exception of muscovite absence (denoted by (Ms)) in a high-temperature field of Vanuatu, muscovite + quartz/coesite + fluid is present in all phase fields. The shading denotes the variance of the phase fields. Univariant phase fields are denoted by thick lines. The semi-parallel thickest curves are geotherms for southeastern (right) and northwestern (left) Japan^12^. The subarc depth range is from ref. 18. Assemblages in the small phase fields below ∼1.5 GPa are omitted for clarity.

For each marine sediment bulk composition, the corresponding phase diagram section (Fig. 1) was computed as a function of pressure (*P*) and temperature (*T*) by free-energy minimization^9^. The thermodynamic database of ref. 10 was used for the properties of all end-member species, and mineral solutions were modelled as described elsewhere^9^. Thermodynamic data for H_2_O, CO_2_ and their mixtures were computed from the equation of state given in ref. 11. To track metamorphic devolatilization along the top of subducted slabs, we adopted the geotherms^12^ for the subduction zones of northwestern and southeastern Japan (Fig. 1). These geotherms are reasonable approximations for the respective extremal low-temperature and high-temperature geotherms for subduction zones (S. M. Peacock, personal communication).

**Figure 2:**
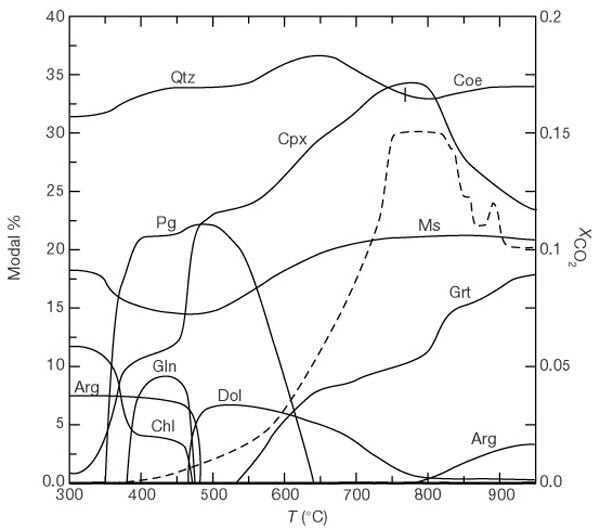
Modal percentages of minerals and fluid composition. Modal percentages of minerals (left ordinate and solid lines) and fluid composition (right ordinate and dashed line) are shown for the average marine sediment bulk composition (‘GLOSS’ in ref. 8) along the high-temperature geotherm shown in Fig. 1. Phase abbreviations as in Fig. 1. The vertical line at ∼770 °C marks the quartz–coesite equilibrium.

Because of compositional degrees of freedom in the crystalline and fluid phases, the phase diagram sections are dominated by multivariant phase fields. Consequently, both mineral modes and compositions vary continuously along geotherms (Fig. 2). Phase relations along geotherms up to a pressure *P* ≈ 3 GPa change significantly (Fig. 1) because of intersection with numerous phase field boundaries. However, at *P* > 3 GPa, the geotherms are subparallel to the phase field boundaries (Fig. 1); consequently, little reaction occurs along geotherms at *P* > 3 GPa. The differences between these regimes are illustrated in Fig. 2. Accordingly, significant changes in the mineralogy and mineral proportions occur up to ∼800 °C (*P* ≈ 3 GPa ) whereas there is comparatively little variation above ∼800 °C.

**Figure 3:**
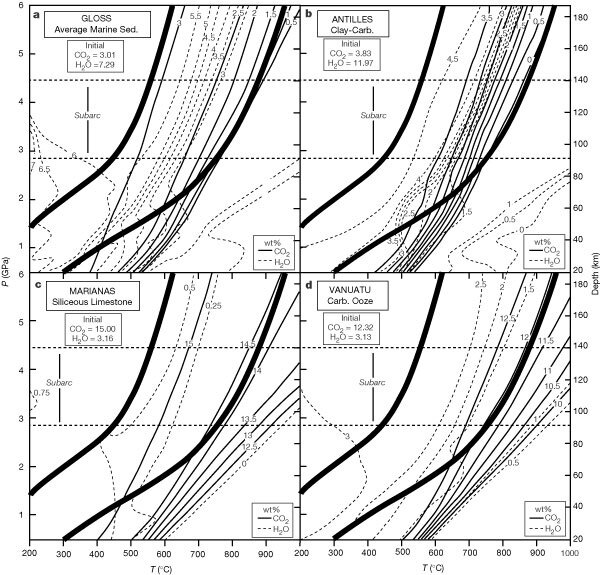
Weight percentages of CO_2_ and H_2_O for selected marine sediment bulk rock compositions (see Fig. 1). **a**, Gloss; **b**, Antilles; **c** Marianas; **d**, Vanuatu. Heavy curved lines are limiting geotherms (see Fig. 1). Values of the initial wt% CO_2_ and H_2_O are given in the insets (from ref. 8). The CO_2_ and H_2_O contents of the fluid phase can be determined by subtracting the data in these diagrams from the initial volatile contents of the protoliths.

The fluid composition (Fig. 2) is controlled by multivariant equilibria involving carbonates and hydrous phases. The rise in the mole fraction of CO_2_, $X_{{\text{CO}}_{\text{2}}}$, up to ∼750 °C correlates with consumption of carbonates (Fig. 2), whereas the diminution in $X_{{\text{CO}}_{\text{2}}}$above ∼750 °C occurs because of aragonite production. To track loss of volatiles we computed the percentage (by weight; wt%) of H_2_O and CO_2_ for carbonate-bearing marine sediments as a function of pressure and temperature (Fig. 3). In the lower-pressure half of Fig. 3, the negative *P–T* slopes of the wt% H_2_O isopleths reflect negative slopes of phase field boundaries (Fig. 1). In contrast to isopleths with negative slopes at lower pressures, isopleths are subparallel to geotherms at *P* > 2–3 GPa (Fig. 3).

Siliceous limestones release about 1 wt% CO_2_ and 1 wt% H_2_O along the high-temperature geotherm (Fig. 3c, d). Because less CO_2_ and H_2_O are released along lower-temperature geotherms, most of the volatile content of siliceous limestones is retained to depths of 180 km and thus such lithologies would undergo little devolatilization upon subduction. This conclusion is compatible with the existence of ultrahigh-pressure marbles^13^.

In contrast with siliceous limestones, H_2_O-rich lithologies with low initial carbonate contents (that is, clay-rich marls) are predicted to undergo considerably more devolatilization (Fig. 3b). Along the high-temperature geotherm, all of the initial CO_2_ (4 wt%) and most of the initial H_2_O (10–11 wt%) is released by 90 km depth (that is, forearcs). For geotherms in the lower-temperature half of the area bounded by the limiting geotherms (Fig. 3b), relatively little CO_2_ and H_2_O would be released. For various geotherms in the higher-temperature half of the area bounded by the limiting geotherms (Fig. 3b), there are significant differences in the amount of devolatilization.

For the carbonate-bearing protoliths considered here, the geotherms at 80–180 km are subparallel to the H_2_O and CO_2_ isopleths (Fig. 3); thus, little or no devolatilization is expected. Consequently, for closed-system behaviour, subducted carbonate-bearing marine sediments would not provide a source of volatiles for arc magmatism. However, decarbonation of marine sediments at these depths may be driven by infiltration of H_2_O-rich fluids originating from intercalated hydrous pelagic or terrigenous sediments, and/or metabasalts in the subjacent slab. Computed^7^ and experimentally determined^14^ high-pressure phase equilibria imply that significant proportions of the initial H_2_O in subducted oceanic metabasalts are released under forearcs and subarcs. Infiltration of the evolved fluid into the overlying subducted sediments would induce decarbonation. But because there are no major dehydration ‘pulses’ in subducted metabasalts under volcanic arcs^7,14^, no corresponding pervasive infiltration of water from dehydrating metabasalts is expected in subarcs. Barring extensive infiltration of externally derived fluids, our study implies marked devolatilization under forearcs (for clay-rich marls with high-temperature geotherms) or retention of H_2_O and CO_2_ to depths well beyond subarcs (for siliceous limestones in all geotherms and clay-rich marls with low-temperature geotherms). Accordingly, most of the initial CO_2_ and H_2_O in subducted marine sediments will not be released beneath volcanic arcs. This inference is consistent with both the deficiency in the amount of CO_2_ released from arc volcanoes compared to the amount of CO_2_ contained within subducted carbonates (Table 1) and with the imbalance between subducted versus expelled H_2_O (ref. 1).

**Table 1:**
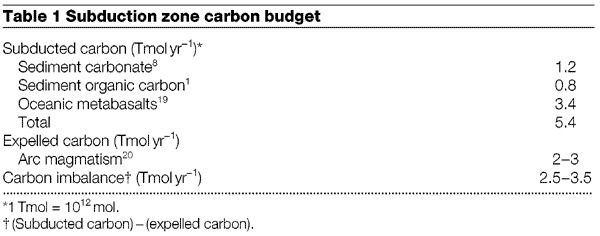
Subduction zone carbon budget

Our equilibrium analysis implicitly assumes that there is no significant kinetic overstepping and metastability of metamorphic reactions. Although significant disequilibrium has been suggested for the transformation of anhydrous oceanic basalts and gabbros to eclogites^15^, the catalytic effect of H_2_O (ref. 15) implies that equilibrium is more likely in dehydrating systems such as subducted sediments.

Melting is an alternative mechanism for release of volatiles from subducted sediment. Recent experiments using marine red clay^16^ suggest that sediment melting does not occur for the geotherms that we consider here. However, because metastable starting materials (for example, red clay) are unsuitable models for subduction-zone metamorphism and melting, confirmation of this conclusion requires experiments with more realistic initial mineral assemblages. Dissolution of minerals in supercritical fluids remains a possible, albeit largely unquantified, alternative mechanism for devolatilization.

As shown in Fig. 2, fluids produced by metamorphism of subducted marine sediments are H_2_O-rich. Consequently, expulsion of such fluids to the overlying mantle wedge would not substantially affect the *P*–*T* conditions of melting (solidus) of the mantle wedge compared to those expected in the presence of a pure H_2_O fluid.

Devolatilization of subducted sediment could contribute to seismicity along the tops of subducted slabs. The continuous nature of devolatilization is compatible with the spread of earthquake hypocentres along individual subduction zones^17^. However, correlation of slab seismicity with metamorphic devolatilization of subducted sediments needs to consider the marked differences in devolatilization for different bulk compositions and geotherms.

## Supplementary information

**TABLE 2:** Bulk Oxide Compositions (wt. %) of Marine Sediments

Oxide	GLOSS*	Antilles**	Marianas***	Vanuatu****
SiO_2_	58.57	46.57	56.53	44.76
TiO_2_	0.62	0.70	0.233	0.365
Al_2_O_3_	11.91	17.99	2.30	11.16
FeO	5.21	7.38	1.56	3.13
MnO	0.32	0.17	0.03	0.11
MgO	2.48	1.61	0.81	1.59
CaO	5.95	5.45	19.23	18.58
Na_2_O	2.43	1.91	0.47	3.58
K_2_O	2.04	2.83	0.60	0.95
P_2_O_5_	0.19	0.08	0.084	0.126
CO_2_	3.01	3.83	15.00	12.32
H_2_O	7.29	11.97	3.16	3.13

All bulk compositions are from Table 1 of Plank and Langmuir^8^.

* Average marine sediment bulk composition ("GLOSS") in Table 3 of Plank and Langmuir^8^.

** Designated "Ant Fe-cc clay" in Table 1 of Plank and Langmuir^8^.

*** Designated "Marianas Chert-Lst" in Table 1 of Plank and Langmuir^8^.

**** Designated "Van Nanno" in Table 1 of Plank and Langmuir^8^
